# Meta-learning for transformer-based prediction of potent compounds

**DOI:** 10.1038/s41598-023-43046-5

**Published:** 2023-09-26

**Authors:** Hengwei Chen, Jürgen Bajorath

**Affiliations:** Department of Life Science Informatics and Data Science, B-IT, Lamarr Institute for Machine Learning and Artificial Intelligence, LIMES Program Unit Chemical Biology and Medicinal Chemistry, Rheinische Friedrich-Wilhelms-Universität, Friedrich-Hirzebruch-Allee 5/6, 53115 Bonn, Germany

**Keywords:** Cheminformatics, Computational chemistry

## Abstract

For many machine learning applications in drug discovery, only limited amounts of training data are available. This typically applies to compound design and activity prediction and often restricts machine learning, especially deep learning. For low-data applications, specialized learning strategies can be considered to limit required training data. Among these is meta-learning that attempts to enable learning in low-data regimes by combining outputs of different models and utilizing meta-data from these predictions. However, in drug discovery settings, meta-learning is still in its infancy. In this study, we have explored meta-learning for the prediction of potent compounds via generative design using transformer models. For different activity classes, meta-learning models were derived to predict highly potent compounds from weakly potent templates in the presence of varying amounts of fine-tuning data and compared to other transformers developed for this task. Meta-learning consistently led to statistically significant improvements in model performance, in particular, when fine-tuning data were limited. Moreover, meta-learning models generated target compounds with higher potency and larger potency differences between templates and targets than other transformers, indicating their potential for low-data compound design.

## Introduction

Predicting new active compounds is one of the major tasks in computer-aided drug discovery, for which machine learning approaches have been widely applied over the past two decades^1,2^. In recent years, deep learning has also been increasingly applied for compound activity and property predictions^1,2^. The prediction of compounds exhibiting a desired biological activity (that is, activity against a target of interest) is mostly attempted using machine learning models for binary classification (that is, a compound is predicted to have or not to have a specific activity)^3–5^. For this purpose, models for class label prediction (active versus inactive compounds) are typically derived based on training sets of known specifically active compounds and randomly selected compounds assumed to be inactive. These qualitative activity predictions mostly involve virtual screening of compound databases to identify new hits. In addition to qualitative predictions of biological activity, predicting compounds that are highly potent against a given target also is of interest. Compound potency prediction can be quantitative or semi-quantitative in nature. Quantitative predictions aim to specify numerical potency values using, for example, quantitative structure–activity relationship (QSAR)^6,7^ or free energy methods^8,9^. Different from qualitative predictions and virtual screening, quantitative potency predictions are usually carried out for small compound sets or structural analogues from lead series. Furthermore, semi-quantitative approaches aim to predict new potent compounds, that is, compounds having higher potency than known actives. For example, such predictions might focus on activity cliffs^10^, which are defined as pairs of structurally similar compounds or structural analogues with large potency differences^10^. Prediction of activity cliffs fall outside the applicability domain of standard QSAR methods^4^.

While quantitative potency predictions are widely carried out, they are difficult to evaluate in benchmark settings. It has been observed that benchmark predictions of different machine learning models and randomized predictions are typically only separated by small error margins^11^, which makes it difficult to non-ambiguously assess relative method performance^11^. Therefore, we currently prefer semi-quantitative approaches focusing on the prediction of potent compounds (rather than trying to predict compound potency values across wide potency ranges). Semi-quantitative predictions can be attempted by deep generative modeling^2^. For example, transformer models have been derived based on pairs of active structural analogues with varying potency to predict activity cliffs and design potent compounds^12,13^. Therefore, the transformer models were conditioned on observed potency differences. This generative design approach successfully reproduced highly potent compounds for different activity classes based on weakly potent input compounds^13^. Transformer models have also been derived for other compound property predictions^14–16^ and generative compound design applications^17–19^ as well as for the prediction of drug-target interactions^20–22^.

Notably, all compound activity and potency predictions depend on available data for learning. Like many other data in early-phase drug discovery, high-quality compound potency measurements for given targets are generally sparse, which limits generative design. Therefore, we are considering machine learning approaches for low-data regimes to enable predictions of potent compounds for targets, for which only little compound data is available. Among learning strategies for sparsely distributed data, active learning^23,24^ and transfer learning^25,26^ have been investigated for machine learning in drug discovery in various studies^24,26^. Transfer learning attempts to use information obtained from related prediction tasks to streamline model derivation for such tasks, while active learning focuses on the selection of most informative training instances for iterative model building. Meta-learning including few-shot learning represents another low-data approach that is relevant for drug discovery^27–30^. In artificial intelligence, meta-learning is a sub-discipline of machine learning^27^. It aims to combine the output of different machine learning models and/or meta-data from these models such as parameters derived from training instances to generate models for other prediction tasks^27^. Alternatively, the same algorithm might be applied to generate models for individual prediction tasks whose outputs are then used to iteratively update a meta-learning model. Hence, meta-learning can also be regarded as a form of ensemble learning. The general aim of meta-learning is achieving transferability of models to related prediction tasks, including the application of prior model knowledge to limit the number of training instances required for new tasks. Given the use of meta-data for learning, the approach is well-suited for parameter-rich deep learning architectures^28^ and -compared to transfer learning- principally applicable to a wider spectrum of predictions tasks. However, in compound design and property prediction, the exploration of meta-learning is still in its early stages. Therefore, we have explored meta-learning in semi-quantitative potency predictions. To this end, we have adapted a transformer architecture designed for the prediction of potent compounds^13^ as a base model for deriving meta-learning models and assessed the potential of meta-learning for predicting highly potent compounds for different activity classes and varying amounts of training data.

## Methods

### Compounds, activity data, and analogue series

Bioactive compounds with high-confidence activity data were collected from ChEMBL (release 29)^31^. Only compounds with direct interactions (assay relationship type: "D") with human targets at the highest assay confidence level (assay confidence score 9) were considered. In addition, potency measurements were restricted to numerically specified equilibrium constants (K_i_ values), which were recorded as (negative decadic logarithmic) pK_i_ values. When multiple measurements were available for the same compound, the geometric mean was calculated as the final potency annotation, provided all values fell within the same order of magnitude. If not, the compound was disregarded. Qualifying compounds were organized into target-based activity classes.

In activity classes, analogue series (AS) with one to five substitution sites were identified using the compound-core relationship (CCR) algorithm^32^. The core structure of an AS was required to consist of at least twice the number of non-hydrogen atoms as the combined substituents. For each AS, all possible pairs of analogues were generated, termed All_CCR pairs. For each activity class, ALL_CCR pairs from all AS were pooled. All_CCR pairs were then divided into CCR pairs with a potency difference of less than 100-fold and activity cliff (AC)*-*CCR pairs with a potency difference of at least 100-fold.

**Figure 1:**
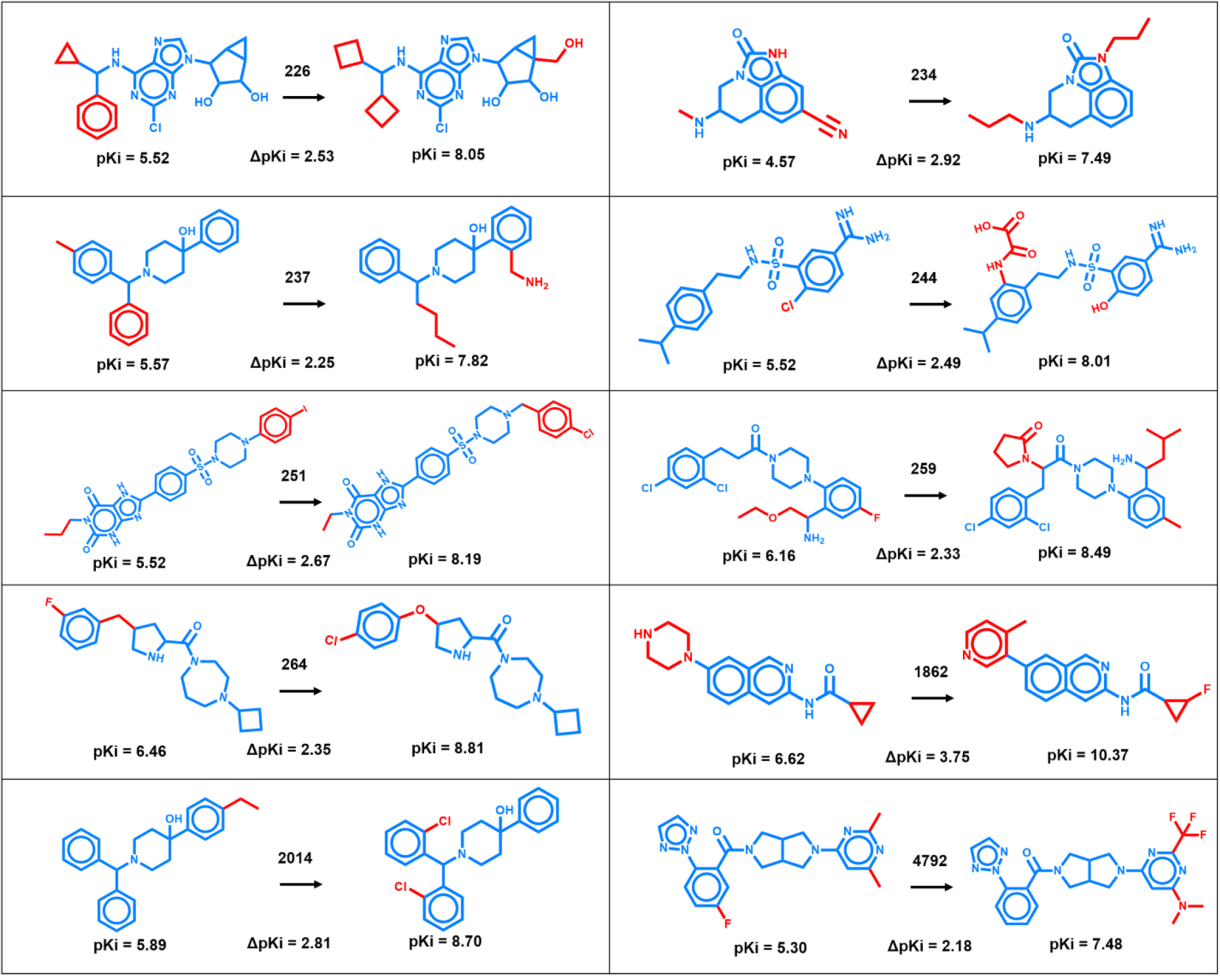
Analogue pairs representing activity cliffs. For each activity class, exemplary AC_CCR pairs are shown and their potency differences are reported. Numbers on arrows identify activity classes according to Table 1. Core structures and substituents are colored blue and red, respectively.

On the basis of the specified data curation criteria and AS distributions, 10 activity classes were assembled that consisted of at least ~ 500 qualifying compounds and ~ 50 AS, as summarized in Table 1. These activity classes included ligands of various G protein coupled receptors and inhibitors of different enzymes. Figure 1 shows exemplary AC_CCR pairs for each class.

**Table 1:** Activity classes.

ChEMBL ID	Target name	Compounds	AS	CCR pairs	AC-CCR pairs
226	Adenosine A1 receptor	1924	318	18,623	1207
234	Dopamine D3 receptor	1529	213	21,008	755
237	Kappa opioid receptor	940	129	19,277	2897
244	Coagulation factor X	702	92	9718	1288
251	Adenosine A2a receptor	1825	312	16,084	870
259	Melanocortin receptor 4	543	145	25,126	3086
264	Histamine H3 receptor	1235	173	10,812	532
1862	Tyrosine-protein kinase ABL	499	64	15,573	1873
2014	Nociceptin receptor	512	52	11,472	1058
4792	Orexin receptor 2	1133	131	12,368	1271

The composition of activity classes is summarized. For each class, the ChEMBL target ID and target name are provided.

### Meta-learning approach

The basic premise of meta-learning, as investigated herein, is parameterizing a model on a series of training tasks by combining and updating parameter settings across individual tasks. This process aims to improve the ability of the model to adapt to new prediction tasks through the use of meta-data.

For designing the meta-learning module of Meta-CLM, we adopted the model-agnostic meta-learning (MAML) framework^28^ for an activity class-specific prediction task distribution *p(T).* Given its model-agnostic nature, the only assumption underlying the MAML approach is that a given model is parameterized using a parameter vector *θ*. Accordingly, a meta-learning model is considered as a function *f*_*θ*_ with parameter vector *θ*. The model aims to learn parameter settings *θ*_*meta*_ that are derived for individual training tasks and updated across different tasks such that they can be effectively adjusted to new prediction tasks. Therefore, for each of a series of prediction tasks, training data are randomly divided into a support set and a query set Accordingly, when the meta-learning module is applied to a new prediction task *T*_*i*_ such as an activity class the current parameter vector *θ*_*meta*_ is updated for task *T*_*i*_ with activity class-specific parameters *θ*_*i*_ obtained by gradient descent optimization minimizing training errors.

**Figure 2:**
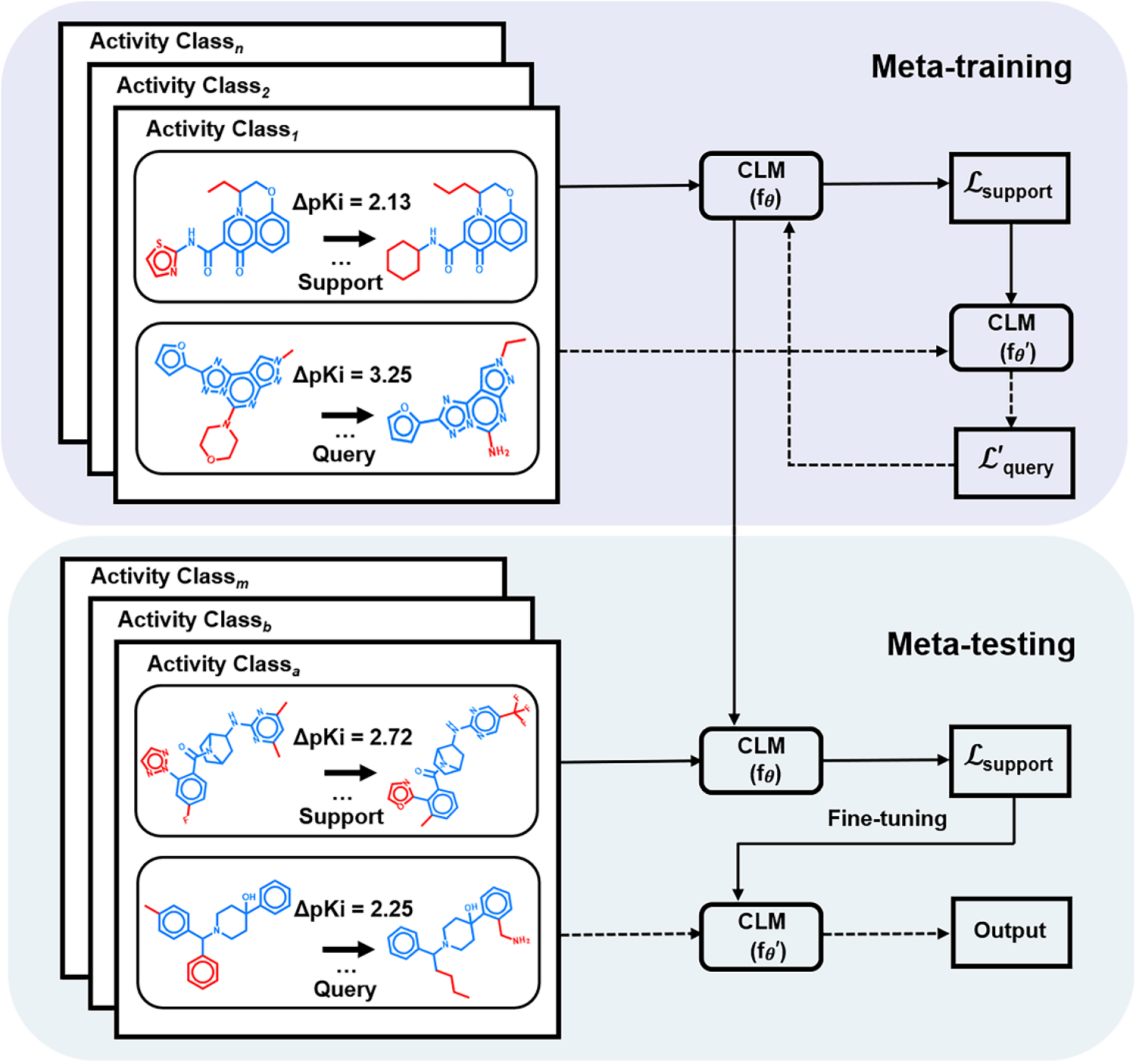
Meta-learning. The illustration summarizes training, fine-tuning, and testing of the meta-learning module of Meta-CLM using exemplary AC-CCR pairs. For each activity class, the support set is used for the initial parameterization of the model (θ). The support loss $\mathscr{L}$_support_ is calculated for updating model parameters (θ′). Then, the query set is used to calculate the prediction loss ${\mathscr{L}}$^′^_query_ for this task. The process is repeated for all training classes, followed by summation of $\mathscr{L}$^′^_query_ over all tasks to further adjust the parameter settings. The trained module then enters the fine-tuning and testing phase. Solid and dashed lines indicate inner and outer loops, respectively, for meta-training and -testing including fine-tuning.

During meta-training, as summarized in Fig. 2, the model *f*_*θ*_ is first updated to a task-specific model *f*_*θ*_′ using its support set. Then, the corresponding query set is used to determine the prediction loss of model *f*_*θ*_′ for this task. The procedure is repeated for all prediction tasks (activity classes). Finally, model parameters are further adjusted for testing by minimizing the sum of the prediction loss over all activity classes. Model derivation based on the support sets and evaluation based on query sets are implemented as inner and outer loops, respectively. For meta-testing, the trained meta-learning module is fine-tuned on a specific activity class, for which parameters are adjusted, as also illustrated in Fig. 2. For each class, an individual fine-tuned model is generated.

The meta-learning process aims to capture prior training information through initial parameter vector adjustments, followed by updates through monitoring of the joint loss across all training tasks^29^. Capturing prior training knowledge should enable the model to more effectively adapt to new prediction tasks based on advanced parameter settings available for initialization and shorter optimization paths with reduced training data requirments^33,34^.

This algorithmic approach differs from conventional multi-task learning where a single model is trained on multiple tasks, aiming to share representations and knowledge between these tasks to collectively improve the basis for learning. Hence, the primary goal of multi-task learning is to improve predictive performance for all tasks by leveraging commonalities between them. Accordingly, model weights are updated based on a combination of the losses from all tasks in a single optimization step. Shared representations for multiple tasks support the model’s ability to simultaneously learn features common to these tasks.

### Transformer models

#### Base model

**Figure 3:**
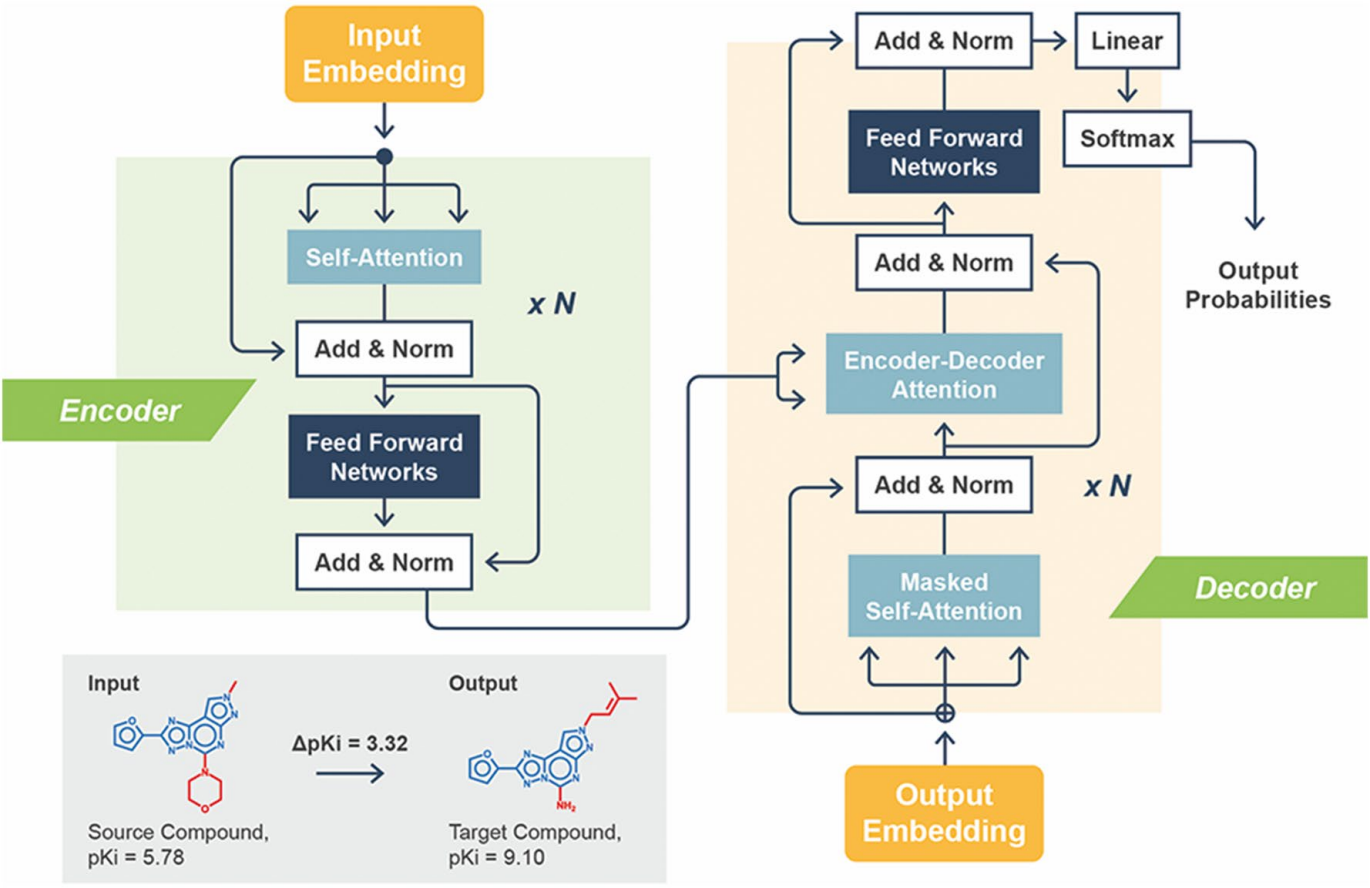
Base CLM. The architecture of the base CLM for designing potent compounds is schematically illustrated (the representation was adapted from ref. 13).

For meta-learning, the transformer architecture derived previously for the prediction of highly potent compounds based on weakly potent templates was adopted^13^. Figure 3 illustrates the architecture of the base CLM. The transformer consisted of multiple encoder-decoder modules with attention mechanism^35^ and was designed for translating string-based representations of chemical structure. Accordingly, the transformer can be perceived as a chemical language model (CLM). The base model (referred to as CLM in the following) was devised to predict compounds with higher potency for given input compounds^13^. An encoder module consisted of encoding sub-layers including a multi-head self-attention sub-layer and a fully connected feed-forward network sub-layer. The encoder compressed an input sequence into a context vector in its final hidden state, providing the input for the decoder module composed of a feed-forward sub-layer and two multi-head attention sub-layers. The decoder transformed the context vector into a sequence of tokens. Both the encoder and decoder modules utilized the attention mechanism during training to effectively learn from the underlying feature space.

During training, the CLM was challenged to learn mappings of template/source compounds (SCs) to target compounds (TCs) conditioned on potency differences (ΔPot) resulting from replacements of substituent(s):

$$\left( {SC,\Delta Pot} \right) \to \left( {TC} \right).$$

Hence, training focused on structural analogues with specific potency differences. Then, given a new *(SC,* Δ*Pot)* test instance, the model generated a set of structurally related TCs with putatively higher potency than SCs.

For transformer modeling, compounds and potency differences must be tokenized. Accordingly, compounds were represented as molecular-input line-entry system (SMILES) strings^36^ generated using RDKit^37^. Tokenization was facilitated by representing atoms with single-character tokens (e.g., "C"or "N"), two-character tokens (e.g., "Cl" or "Br"), or tokens enclosed in brackets (e.g. "[nH]" or "[O-]"). Potency differences were subjected to binning tokenization^12,13,38,39^ by dividing the global range of potency differences (-6.62 to 6.52 pK_i_ units) into 1314 bins with a constant width of 0.01. Each bin was encoded by a single token and each potency difference was assigned to the corresponding token^12,13^. In addition, two special "start" and "end" tokens were defined as the start and end points of a sequence, respectively.

The model was pre-trained using a large set of 881,990 All_CCR pairs originating from 496 public activity classes^13^. For pre-training, All_CCR triples *(Cpd*_*A*_*, Cpd*_*B*_*, Pot*_*B*_*-Pot*_*A*_*)* were generated in in which *Cpd*_*A*_ and *Cpd*_*B*_ represented the SC and TC, respectively, and (*Pot*_*B*_*-Pot*_*A*_) their potency difference.

CLM was implemented using Pytorch^40^. Default hyperparameter settings were used for the transformer architecture together with a batch size of 64, learning rate of 0.001, and encoding dimension of 256. During training, the transformer model minimized the cross-entropy loss between the ground-truth and output sequence. The Adam optimizer was used^41^. The model was trained for a maximum of 1000 epochs. At each epoch, a checkpoint was saved, and the final model was selected based on the minimal loss.

The base model achieved a reproducibility of 0.857 for the entire test set (corresponding to 10% of pre-training set). Hence, the base CLM model regenerated ~ 86% of the target compounds from CCR-triples not used for training.

#### Model for meta-learning

The CLM variant for meta-learning was also implemented using Pytorch following the protocol described above. The meta-learning model, designated Meta-CLM, consisted of two modules including the base model for generating mappings of SCs to TCs conditioned on potency differences and the meta-learning module (the design of which is detailed below). For derivation of the metal-learning module, a subset of 176 of the 496 activity classes was selected for which at least 300 All-CCR pairs per class were available, amounting to a total of 491,688 qualifying All_CCR triples. For meta-learning, each activity class was considered a separate training task (see below). Therefore, All_CCR triples from each class were randomly split into support set (80%) and query set (20%). The Adam optimizer was used for gradient descent optimization during meta-learning.

#### Model fine-tuning

For fine-tuning and comparative evaluation of CLM and Meta-CLM, the 10 activity classes in Table 1 were used. Fine-tuning was separately carried out using AC-CCR pairs from each class. The AC-CCR pairs from each class were randomly divided into fine-tuning (80%) and test instances (20%). In each case, it was confirmed that the fine-tuning and test pairs had no core structure overlap (otherwise, a new partition was generated). For fine-tuning, AC_CCR pairs were exclusively used. AC_CCR triples were ordered such that TC was the highly potent compound. To assess the ability of CLM and Meta-CLM to learn in low-data regimes, model variants were derived based on 10%, 25%, 50% and 100% of the training data. To adapt to differently sized training sets, the pre-trained model was fine-tuned with a smaller learning rate of 0.0001. With a maximum of 200 training epochs, the final fine-tuned model was selected based on minimal cross-entropy loss.

#### Model evaluation

For each activity class, CCR pairs sharing core structures with the fine-tuning set were excluded, then the final test set was generated by adding the remaining CCR pairs to test AC-CCR pairs. Test set CCR and AC-CCR pairs yielded class-dependent numbers of unique CCR and AC-CCR test compounds. To evaluate the performance of each fine-tuned CLM and corresponding Meta-CLM, test compounds were divided into two categories: SCs with a maximum potency of 1 μM (corresponding to a pK_i_ value of 6) and TCs with a potency greater than 1 μMol (pK_i_ > 6). These test TCs were termed known target compounds (KTCs), which represented highly potent test compounds. Table 2 reports the test composition for each activity class. Depending on the activity class, 139 to 3838 KTCs were available.

**Table 2:** Test sets.

ChEMBL ID	CCR Pairs	Unique CCR CPDs	AC-CCR Pairs	Unique AC-CCR CPDs	Overlapping CPDs	SCs (pk_i_ < = 6)	KTCs (pk_i_ > 6)
226	5950	1174	144	84	80	359	819
234	7790	913	50	53	53	89	824
237	1032	477	31	24	20	115	366
244	1949	308	287	118	88	90	248
251	4706	5210	85	57	38	1391	3838
259	702	169	59	69	33	66	139
264	4756	840	72	81	58	33	830
1862	4554	175	82	51	51	27	148
2014	1388	256	80	62	29	23	266
4792	1941	615	49	50	48	146	471

For each activity class (ChEMBL IDs are used according to Table 1), the composition of the test set is reported. CPD stands for compound.

For each test set SC, 50 hypothetical TCs were sampled and compared to available KTCs. The ability of a model to reproduce KTCs was considered as the key criterion for model validation.

## Results

### Reproducibility of known target compounds

We first analyzed the ability of Meta-CLM to reproduce KTCs in comparison to CLM. The results are reported in Table 3. For all activity classes, Meta-CLM and CLM correctly reproduced multiple KTCs over all fine-tuning conditions, thus providing non-ambiguous proof for the models’ ability to predict potent compounds. From correctly predicted SC-KTC pairs, unique KTCs were extracted (a given KTC can occur in multiple pairs). The number of correctly predicted SC-KTC pairs and unique KTCs varied depending on the activity class. Importantly, Meta-CLM consistently predicted more SC-KTC pairs and unique KTCs than CLM across all activity classes, without an exception. For Meta-CLM, the number of SC-KTC pairs varied from 71 to 5102 pairs when utilizing 100% of the training samples and the number of unique KTCs varied from 27 to 287, corresponding to a reproducibility ratio of ~ 7% to ~ 45% of available KTCs per class. For comparison, CLM, the base model, generated from 53 to 4385 SC-KTC pairs, with 23 to 241 unique KTCs and a corresponding reproducibility ratio of ~ 5% to ~ 36% per class. Moreover, for decreasing numbers of fine-tuning samples, Meta-CLM consistently reproduced more KTCs than CLM. For complete fine-tuning sets, Meta-CLM and CLM reached mean reproducibility rates of ~ 21% and ~ 14%, respectively. For only 10% of the fine-tuning samples, Meta-CLM reached a mean reproducibility rate of ~ 15% compared to only ~ 7% for CLM. Thus, Meta-CLM learned more effectively from sparse data than CLM, consistent with the aims of meta-learning.

**Table 3:** Reproducibility of compound pairs and known target compounds.

ChEMBL ID	Ratio	SC-KTC Pairs		Unique KTCs		Reproducibility (%)	
		Meta-CLM	CLM	Meta-CLM	CLM	Meta-CLM	CLM
226	10	799	379	223	118	27.2	14.4
	25	965	510	263	167	32.1	20.4
	50	1041	614	268	183	32.7	22.3
	100	1193	735	287	216	35.0	26.4
234	10	174	75	50	19	6.1	2.3
	25	268	130	68	36	8.3	4.4
	50	343	197	87	58	10.6	7.0
	100	398	239	101	71	12.3	8.6
237	10	397	325	90	52	24.6	14.2
	25	449	366	101	66	27.6	18.0
	50	433	362	103	81	28.1	22.1
	100	480	429	118	102	32.2	27.9
244	10	109	62	26	11	10.5	4.4
	25	111	66	31	17	12.5	6.9
	50	160	98	39	28	15.7	11.3
	100	193	129	45	36	18.2	14.5
251	10	3930	3288	233	138	6.1	3.6
	25	4685	3959	249	172	6.5	4.5
	50	4856	4153	245	201	6.4	5.2
	100	5102	4385	264	241	6.9	6.3
259	10	51	40	14	5	10.1	3.6
	25	73	60	24	13	17.3	9.4
	50	98	88	30	22	21.6	15.8
	100	129	116	33	30	23.7	21.6
264	10	16	11	14	6	1.7	0.7
	25	33	19	28	17	3.3	2.1
	50	54	33	42	31	5.1	3.7
	100	71	53	57	40	6.9	4.8
1862	10	65	29	25	6	16.9	4.0
	25	96	48	28	14	18.9	9.5
	50	93	56	32	23	21.6	15.5
	100	147	94	33	30	22.3	20.3
2014	10	85	53	20	9	7.5	3.4
	25	102	71	25	12	9.4	4.5
	50	113	84	22	16	8.3	6.0
	100	131	99	27	23	10.2	8.7
4792	10	849	622	176	106	37.4	22.5
	25	976	746	179	129	38.0	27.4
	50	1085	828	199	151	42.3	32.1
	100	1262	969	212	170	45.0	36.1

**Figure 4:**
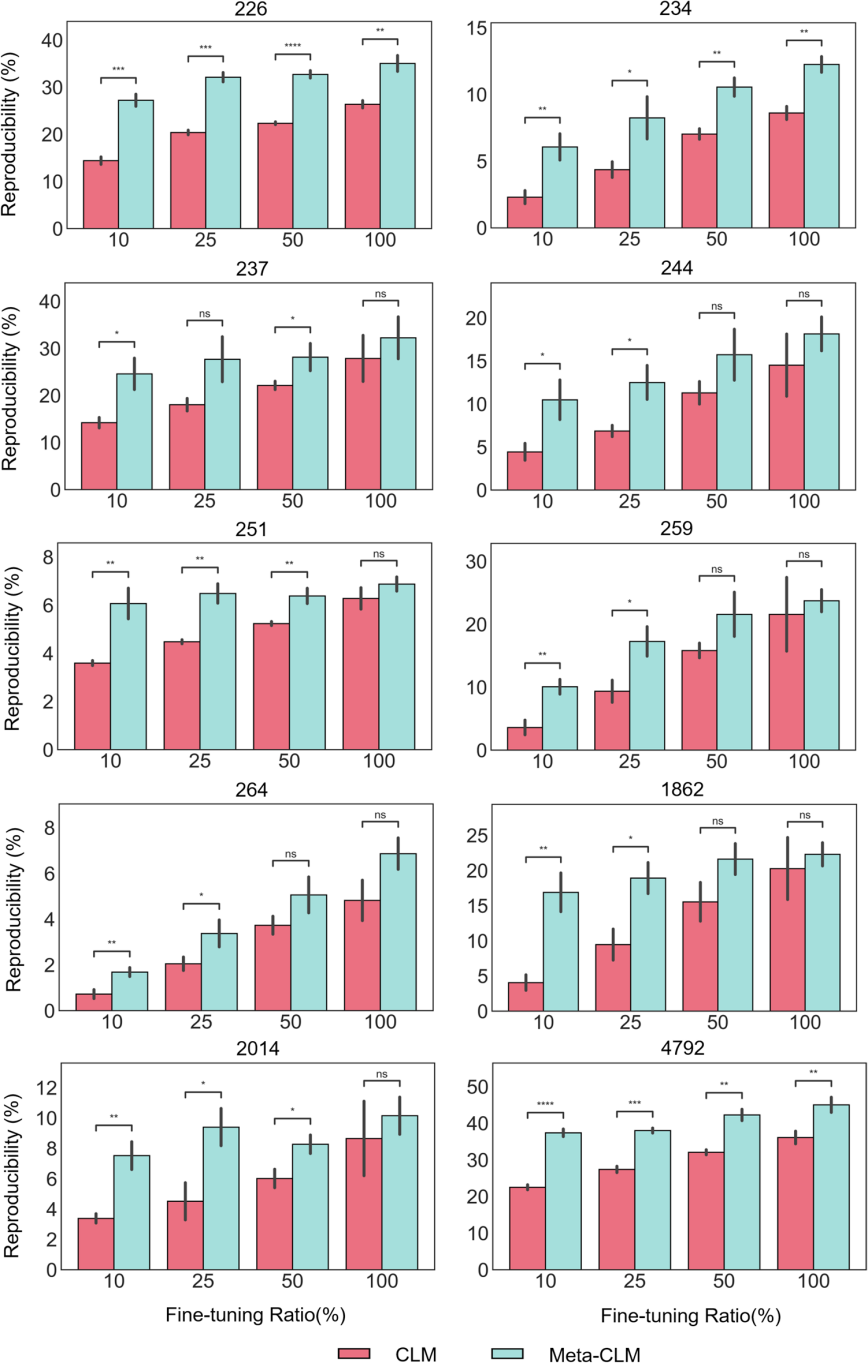
Reproducibility of known target compounds. For each activity class, the proportion of correctly reproduced KTCs is reported for Meta-CLM and CLM over varying percentages of fine-tuning samples. Mean and standard deviations (error bars) are provided. To assess the statistical significance of observed differences between reproducibility rates, independent-samples t tests were conducted: 0.05 < p ≤ 1.00 (ns), 0.01 <  *p* ≤  0.05 (*), 0.001 <  *p*  ≤ 0.01 (**), 0.0001 <  *p*  ≤ 0.001 (***),  *p* ≤ 0.0001 (****). Stars denote increasing levels of statistical significance and “ns” stands for “not significant”.

Figure 4 illustrates the differences in KTC reproducibility rates between Meta-CLM and CLM. Independent-samples t-tests were carried out to assess the statistical significance of the observed differences. For complete fine-tuning sets, increases in reproducibility detected for Meta-CLM were statistically significant for three of 10 activity classes. However, for fine-tuning sets of deceasing size, 25 of 30 increases across all activity classes were statistically significant, thus providing further evidence for the ability of Meta-CLM to more effectively learn from sparse data. For most classes, there was a sharp decline in CLM reproducibility rates when 25% or 10% of the fine-tuning samples were used.

We also note that both models produced large numbers of novel candidate compounds for SCs. For complete fine-tuning sets, Meta-CLM and CLM generated on average 2375 and 2818 new candidate compounds per activity class (ranging from 119 to 9952 and 234 to 10,779 candidates, respectively). While these new compounds cannot be considered for model validation, they provide large pools of candidates for practical applications in the search for potent compounds.

### Compound potency

In addition to reproducing KTCs, the actual potency level of correctly predicted KTCs and potency differences between SCs and corresponding KTCs represented other highly relevant criteria for model assessment. According to our semi-quantitative design approach, ideally, the models should predict highly potent compounds from given SCs. Therefore, we next analyzed the potency of correctly predicted KTCs and potency differences between Meta-CLM and CLM.

#### Known target compounds

**Figure 5:**
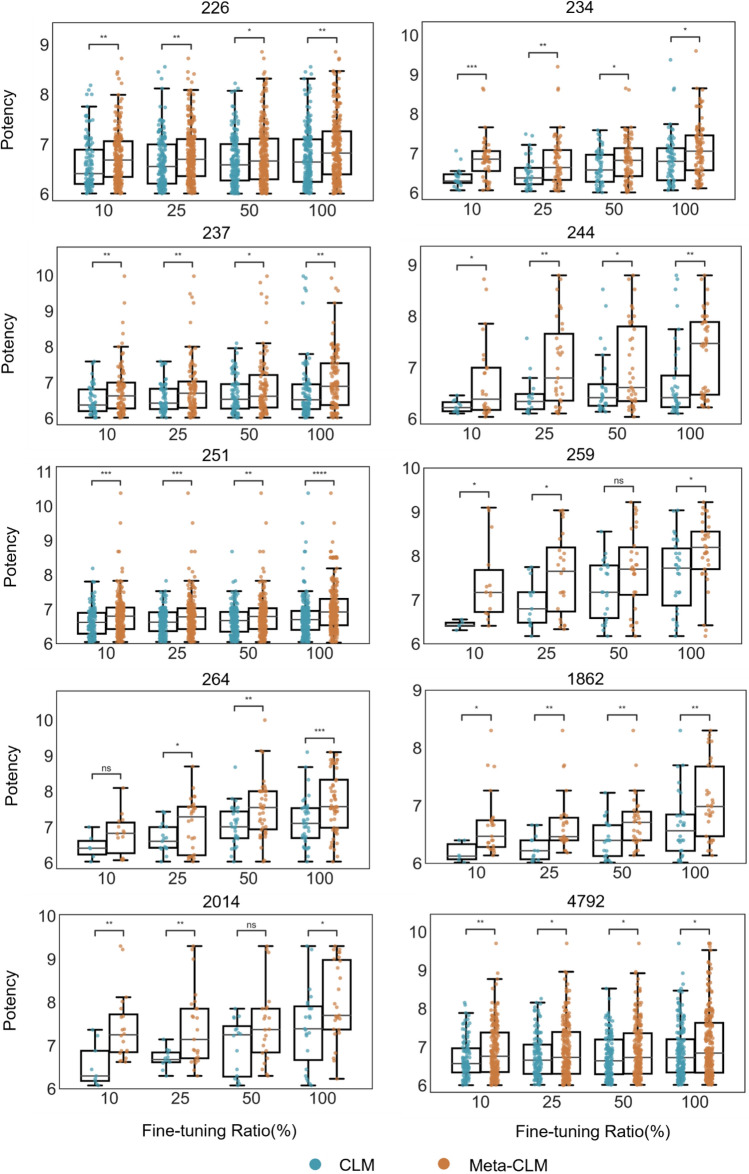
Potency value distribution of reproduced known target compounds. For all activity classes, boxplots report the distributions of logarithmic potency values of KTCs correctly reproduced by Met-CLM and CLM over varying numbers of fine-tuning samples. To assess the statistical significance of differences between potency value distributions, independent-samples t tests were conducted: 0.05 <  *p*  ≤ 1.00 (ns), 0.01 <  *p* ≤ 0.05 (*), 0.001 <  *p* ≤ 0.01 (**), 0.0001 <  *p* ≤ 0.001 (***),  *p*  ≤ 0.0001 (****).

Figure 5 shows the distributions of logarithmic potency values of KTCs reproduced by Meta-CLM and CLM. Importantly, KTCs generated by Meta-CLM were overall consistently more potent than those generated by CLM across all activity classes and fine-tuning conditions. Thirty-eight of the total of 40 observed differences between the respective potency value distributions were statistically significant. Especially for 25% and 10% of the fine-tuning samples, Meta-CLM generated multiple KTCs with low-nanomolar or even sub-nanomolar potency for each activity class, whereas CLM only generated a few KTCs with potency higher than 10 nM (pKi > 8) for three classes.

#### Potency differences between source and target compounds

**Figure 6:**
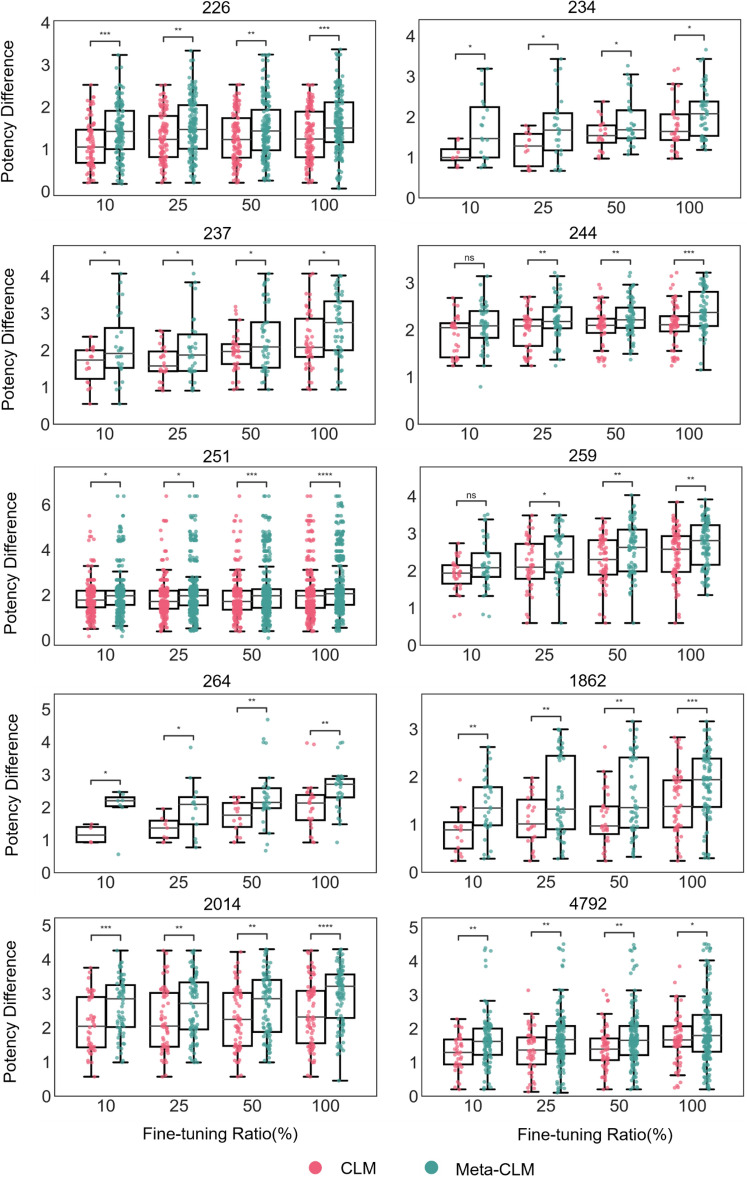
Distribution of potency differences between source and known target compounds. For all activity classes, boxplots report the distributions of logarithmic potency differences for SC-KTC pairs predicted by Meta-CLM and CLM over varying numbers of fine-tuning samples. To assess the statistical significance of differences between the distributions, independent-samples t tests were conducted: 0.05 <  *p* ≤ 1.00 (ns), 0.01 <  *p*  ≤ 0.05 (*), 0.001 <  *p* ≤ 0.01 (**), 0.0001 <  *p* ≤ 0.001 (***),  *p* ≤ 0.0001 (****).

Furthermore, we analyzed potency differences captured by SC-KTC pairs. Following our design strategy, increasingly large potency differences between corresponding SCs and correctly reproduced KTCs were favored. Figure 6 shows the distribution of potency differences between corresponding SCs and KTCs for Meta-CLM and CLM predictions. In the case of Meta-CLM (CLM), four (six) activity classes displayed median potency differences between SCs and corresponding KTCs between one and two orders of magnitude (10- to100-fold) and the remaining six (four) classes displayed median potency differences exceeding two orders of magnitude (> 100-fold) for complete fine-tuning sets. Hence, significant potency differences were generally observed. For half of the activity classes, median potency differences were comparable for all fine-tuning conditions when separately viewed for Meta-CLM and CLM, respectively. However, when Meta-CLM and CLM were compared, potency differences of SC-KTC pairs were consistently larger for Meta-CLM. Again, 38 of 40 observed differences were statistically significant. Overall, many more KTCs with at least 1000-fold higher potency than the corresponding SCs were generated by Meta-CLM compared to CLM. Thus, Meta-CLM predicted KTCs with overall higher potency than CLM and much larger potency differences between SCs and KTCs.

## Conclusion

In this work, we have explored meta-learning for the prediction of potent compounds using conditional transformer models. Compound potency predictions are of high interest in drug discovery but high-quality activity data available for machine learning are typically sparse. For these predictions, meta-learning was of particular interest to us because the approach is well-suited for models that are rich in meta-data, yet currently only little explored for drug discovery applications. Therefore, we have adapted a previously investigated transformer architecture to construct a meta-learning model by adding a special meta-learning module to a pre-trained transformer. Then, meta-learning model variants were derived for different activity classes and their performance in the design of potent compounds was compared to reference transformers. For model validation, the ability to reproduce potent KTCs served as the major criterion. All models successfully reproduced KTCs. However, compared to reference models, meta-learning significantly increased the number of correctly predicted KTCs across all activity classes, especially for decreasing numbers of fine-tuning samples. This was an encouraging finding, consistent with expectations for successful meta-learning. Moreover, meta-learning models also produced target compounds with overall higher potency than other transformers and larger potency differences between templates and targets. These improvements were not anticipated but are highly attractive for practical applications. The generative models designed for predicting potent compounds produced large numbers of candidate compounds with novel structures. New candidate compounds predicted by the meta-learning models should represent an attractive resource for prospective applications in searching for potent compounds for targets of interest. Taken together, the results reported herein, provide proof-of-concept for the potential of meta-learning in generative design of potent compounds. Moreover, in light of our findings, we anticipate that meta-learning will also be a promising approach for other compound design applications in low-data regimes.

## Data Availability

Calculations were carried out using publicly available programs and compound data. Python scripts generated for the study and the activity classes used are available via the following link: https://uni-bonn.sciebo.de/s/kfAQZ0mbCGHtr0m.
